# Creating allies: qualitative exploration of young women’s preferences for PrEP methods and parents’ role in PrEP uptake and user support in urban and rural Zambia

**DOI:** 10.1186/s12905-024-02913-7

**Published:** 2024-01-25

**Authors:** Christina Misa Wong, Tendai Munthali, Featherstone G. Mangunje, Mercy L. Katoka, Holly M. Burke, Bupe Musonda, Musonda Musonda, Catherine S. Todd

**Affiliations:** 1 Global Health & Population, FHI 360, 359 Blackwell Street, Suite 200, Durham, NC 27701 USA; 2 Ministry of Health, Government of the Republic of Zambia, Ndeke House, Haile Selassie Avenue, P.O. Box 30205, Lusaka, Zambia; 3 FHI 360 Zambia, Tiyende Pamodzi Road, Off Nangwenya Road, Farmers Village, Showgrounds Area, P.O. Box 320303, Lusaka, 10101 Zambia; 4 Reproductive, Maternal, Newborn, and Child Health, FHI 360, 359 Blackwell Street, Suite 200, Durham, NC 27701 USA; 5 United States Agency for International Development (USAID), Embassy of the United States of America, Subdivision 694/Stand 100 Ibex Hill Road, P.O. Box 320373, Lusaka, 10101 Zambia

**Keywords:** Adolescent girls and young women, HIV prevention, PrEP, Parental engagement, Parents

## Abstract

**Background:**

Zambian adolescent girls and young women (AGYW) have high HIV incidence and face barriers to the use of pre-exposure prophylaxis (PrEP). Parental support improves PrEP use and adherence in some settings, but negative parental attitudes toward HIV prevention may inhibit engagement with AGYW. We explored perceptions of future PrEP methods among AGYW and parents and parent-youth engagement on HIV prevention and PrEP use.

**Methods:**

We conducted a qualitative descriptive study among AGYW and parents of AGYW in five provinces in Zambia in September–October 2021. We conducted 10 focus group discussions (FGDs) and four in-depth interviews (IDIs) with AGYW participants (*n* = 87) and seven FGDs and four IDIs among parents of AGYW (*n* = 62). All FGDs and IDIs were audio-recorded, transcribed verbatim, and analyzed to identify qualitative themes.

**Results:**

Most AGYW participants preferred the discreet nature and longer duration of injectable PrEP compared to the PrEP ring and oral PrEP. Many AGYWs reported inability to disclose PrEP use to their parents due to lack of parental support based on cultural taboos against premarital sex. Nevertheless, AGYW participants said they would like to talk to their parents about PrEP so their parents could support their use. Many parents also described difficulties discussing PrEP with their daughters because of cultural and religious beliefs about abstinence from sex before marriage. However, parents acknowledged that the threat of HIV is real and said they need PrEP knowledge and guidance on speaking with their children about HIV prevention and PrEP.

**Conclusions:**

Although many parents are currently not playing a role in daughters’ decisions about PrEP use, both parents and AGYW are willing to engage with each other on HIV prevention issues. To foster parent-child engagement, HIV prevention programs should not only provide information about PrEP but also address social norms that impede discussion of HIV prevention and equip both parents and AGYW with skills and support for such conversations. Community sensitization is also needed as new PrEP products are introduced, to create an enabling environment for parent-child engagement by increasing awareness, countering misconceptions, and reducing stigma.

**Supplementary Information:**

The online version contains supplementary material available at 10.1186/s12905-024-02913-7.

## Introduction

HIV incidence has been decreasing overall in sub-Saharan Africa but remains high for adolescent girls and young women (AGYW) ages 15 to 24 years, who represent 10% of the population but 25% of new HIV infections [1]. AGYW are a priority population for HIV prevention, particularly pre-exposure prophylaxis (PrEP), and numerous approaches are implemented to facilitate awareness, uptake, and use of prevention methods [2–5]. In many cases, however, oral PrEP awareness and uptake are high, but longer-term effective use remains a challenge [5–8].

Perceived and actual key influencer opinions are important factors in AGYW’s decisions about initiating and continuing PrEP use [9–12]. Among key influencers, parents and caregivers have a substantial effect on young women’s decisions to access and use HIV prevention methods, particularly among younger adolescents and those living with their parents [11–14]. This influence can be positive — resulting, for example, in higher oral PrEP continuation among AGYW with parental support in South Africa [12, 15] — or negative, associated with poor adherence in an oral PrEP trial [16]. To date, most research on parents and caregivers as key influencers of AGYW for HIV prevention has focused on AGYW’s perspectives, with relatively less inclusion of parents’ insights [12, 17–20].

New PrEP methods — the dapivirine vaginal ring (PrEP ring) and injectable cabotegravir (CAB PrEP) — are entering the market and offer advantages including extended duration of action, freedom from daily dosing, and ability to conceal use, addressing some factors contributing to oral PrEP discontinuation among AGYW [21, 22]. Available data reflect preferences for long-acting methods [21–24], but most of the studies were conducted among AGYW with no prior PrEP experience and before oral PrEP implementation moved to scale in many countries. It is important to understand perspectives on parental engagement, PrEP use disclosure, and support for longer-acting methods to facilitate implementation of new PrEP methods in the context of oral PrEP availability.

Like other countries in eastern and southern Africa, Zambia has an HIV incidence among AGYW ages 15 to 24 years twice that of their male peers, and oral PrEP uptake has been high among AGYW able to access services [25, 26]. However, ongoing effective use among this priority population for HIV prevention is relatively low [26]. As new PrEP method options become available and are considered for introduction in Zambia, a better understanding of AGYW’s HIV prevention method and service delivery preferences is needed. Parents’ perspectives on AGYW’s PrEP use also warrant study. Results will help guide community sensitization and implementation efforts.

The PROMISE collaboration and MOSAIC project, funded by the U.S Agency for International Development (USAID) and President’s Emergency Plan for AIDS Relief (PEPFAR), aim to accelerate introduction of new biomedical HIV prevention products for female populations. In Zambia, the PROMISE collaboration explored the landscape for PrEP ring introduction with activities including a larger qualitative study of stakeholder PrEP method and service delivery preferences in five urban, rural, and transnational border provinces. In this sub-analysis, we summarize parent-youth engagement on HIV prevention from AGYW and parents/caregivers of AGYW to inform AGYW HIV prevention programming in Zambia.

## Methods

### Study overview, design and setting

**Fig. 1:**
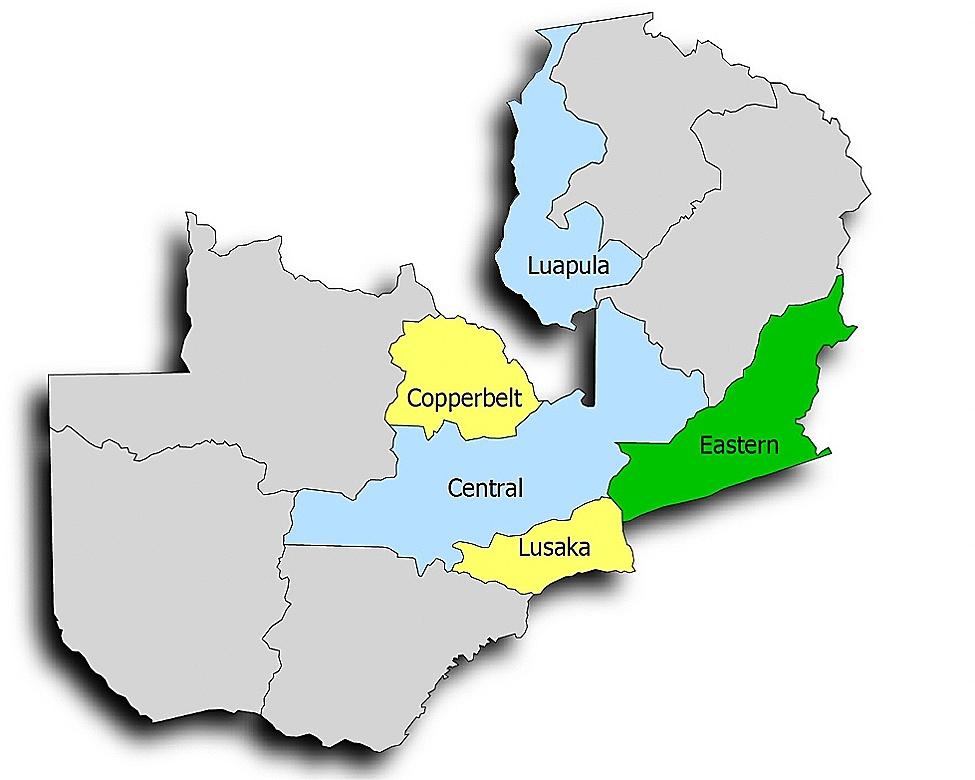
Map of Zambia showing provinces included in the study. Provinces marked in yellow represent urban provinces; blue represents rural provinces and green represents a province with an urban-rural mix with transnational boundaries

In this qualitative descriptive study, focus group discussions (FGDs) and in-depth interviews (IDIs) were conducted among participants from five Zambian provinces representing three geographic groups: (1) rural, (2) urban, and (3) rural-urban mix with transnational borders (Eastern province) (Fig. 1). The “rural-urban mix with transnational borders” area were delineated as a separate group in these specific provinces because they are along major transportation routes where sex work is common and have a different risk context for HIV.

A study advisory board with representatives from the National AIDS Council and civil society organizations (CSOs) provided input on interview guide content, data collection sites, and analysis priorities and facilitated participant recruitment. The National AIDS Council is a parastatal organization that works closely with CSOs and district and community level actors with particular focus on demand creation, community-level prevention, care, and treatment activities. Site selection focused on program sites where many potential participants were accessible, which were predominantly facilities with youth and parent activities or health centers with youth-friendly services.

Parents and youth were recruited predominantly through DREAMS (Determined, Resilient, Empowered, AIDS-free, Mentored, and Safe) program sites (except in Luapula Province, which had no DREAMS sites). DREAMS — PEPFAR’s flagship intervention to prevent HIV among AGYW — includes both AGYW and parent activities in Zambia, aims to facilitate parent-youth engagement on HIV prevention, and offers integrated HIV and sexual and reproductive health (SRH) services, support groups, counseling, and economic literacy and livelihood training activities [27–29]. Prior to data collection, the study team engaged staff from technical assistance projects (e.g., USAID/PEPFAR SAFE, DISCOVER HEALTH, and ACTION HIV) and CSOs, including local CSOs such as Copper Rose Zambia, Marie Stopes Zambia, and the Centre for Infectious Diseases Research in Zambia (CIDRZ), to identify eligible individuals in their programs who could provide insights on topics related to HIV prevention product choice.

### Participant selection

Participants were purposively sampled to include AGYW ages 18 to 22 years speaking the predominant language of the province or English. Prior sexual activity and PrEP use were not included as eligibility criteria. Eligible mothers, fathers, and caregivers were those known to community leaders or program staff as having an adolescent or young adult daughter or dependent living at home who speak the predominant provincial language or English. Identical criteria were used to select FGD and IDI participants.

The goal was to conduct two FGDs for each participant group (AGYW and parents of AGYW) in each of the five provinces, with additional IDIs to help gauge data saturation and explore sensitive topics. (The IDIs confirmed that data saturation had been achieved.) This sample size was guided by research showing that 80% of all themes are discoverable with two to three FGDs per participant group [30]. IDI participation was offered to eligible individuals after the minimum number of FGD participants had been selected. For FGDs with AGYW, we had separate groups for those who had ever used PrEP and those who had never used PrEP. For FGDs with parents, we had mixed-gender groups based on convenience.

### Data collection

IDIs and FGDs were conducted in person by 10 experienced interviewers, including one young person and six consultants who received training that included informed consent, research ethics, and qualitative interviewing techniques. All but one of the AGYW IDIs and FGDs were conducted by a female interviewer, and the interviewers did not have established relationships with participants prior to the study. The study team scheduled IDIs and FGDs at the host public health facilities, CSOs, community centers, or DREAMS program sites convenient to potential participants. They used two semi-structured guides (Table 1), one for AGYW and the other for parents, that had been pretested among small (*n* = 3–5) groups of AGYW and parents.

**Table 1:** Topic domains for IDIs and FGDs with AGYW and parents/caregivers of AGYW

AGYW	Parents/caregivers of AGYW
• Perspectives on HIV prevention and family planning (FP) services • Oral PrEP awareness, opinions, and experiences • Awareness and perceptions about the PrEP ring and injectable cabotegravir (CAB PrEP) • Preferences for PrEP method selection and use • Preferences for PrEP service delivery • Perspectives on parents’ role and engagement in their daughters’ decisions about and use of PrEP and contraceptives • Recommendations for making new PrEP product introduction successful	• Awareness and perceptions about oral PrEP, the PrEP ring, and CAB PrEP • Perceived parental reactions to AGYW’s PrEP use and disclosure • Perspectives on parents’ role and engagement in their daughters’ decisions about and use of PrEP and contraceptives • Recommendations for making new PrEP product introduction successful

Prior to initiating data collection, we screened participants for eligibility, and they provided written informed consent to participate in an audio-recorded IDI or FGD. Participants then completed a brief, tablet-based demographic and health history questionnaire that included questions about ever use of PrEP, smartphone/tablet ownership, and whether they ever accessed health information from the internet. During the IDIs and FGDs, participants received a basic overview of oral PrEP, the PrEP ring, and CAB PrEP methods after interviewers determined their general awareness of these methods (see Supplementary Material 1).

IDIs and FGDs were conducted in English, Bemba, and Nyanja, with IDIs lasting about an hour and FGDs ranging from one to two hours. All data were collected following Zambian national COVID-19 prevention guidelines and precautions; study staff and participants received masks and hand sanitizers, and IDIs and FGDs were conducted outdoors or in well-ventilated areas.

Study team members transcribed the audio recordings using a standardized transcription protocol, with a second team member reviewing transcripts against audio files for quality assurance [31]. When the audio recordings were not in English, they were simultaneously translated and transcribed into English.

### Data analysis

A two-step, rapid qualitative analysis method was used to analyze the data from the FGDs and IDIs [32]. For the first step, we developed a structured template for each of the two participant types (AGYW and parents of AGYW) using Microsoft Excel. This template included columns for each question from the IDI/FGD guides with a row for each transcript. A team of four staff then reviewed each transcript to summarize the responses for each question and recorded them in the appropriate template with relevant illustrative quotes.

For the second step, one analyst reviewed the template entries by question and by participant type to develop summary reports describing themes and sub-themes. We also analyzed and compared data by participant type and geographical area (urban, rural, and Eastern provinces). We also confirmed during data analysis that data saturation had been reached and that there was no need for additional focus group discussions. Sociodemographic data were analyzed descriptively.

During the analysis period, preliminary results were presented at a stakeholder consultation convened in Lusaka, Zambia, July 26–28, 2022, to explore challenges to parent–youth engagement on HIV prevention. Participating stakeholders included representatives of government and parastatal organizations, implementing organizations, CSOs, and advocacy groups; community leaders and members, including youth delegates from each Zambian province; and participants in the AGYW and parent study. Results regarding AGYW preferences among the three PrEP methods and the dynamics of parent and youth engagement were presented, with questions periodically posed to the audience. The audience was specifically asked whether the findings resonated with their experience. We summarized their responses to enrich and informally validate the study results with a broader audience.

### Ethics approval

The activity was determined not to be research by FHI 360’s Office of International Research Ethics (IRBNet ID: 1733984-1) and was approved by the ERES Converge Institutional Review Board (2021-July-003) and National Health Research Authority (NHRA00004/2/08/2021) in Zambia.

## Results

### Participant characteristics

We conducted 10 FGDs and four IDIs with AGYW participants (87 participants) and seven FGDs and four IDIs with parents of AGYW (62 participants). The median age of AGYW participants was 20, and most (90%) had completed secondary school (Table 2). Many (75%) were unmarried with a regular partner, and about half (51%) had ever used oral PrEP. Parent participants were mostly women (74%), with a median age of 42.5, and many had a primary (35%) or secondary school (42%) education. About three-quarters (74%) were married, and slightly less than half (47%) owned a smartphone or tablet.

**Table 2:** Participant sociodemographic characteristics

	AGYW (*n* = 87)		Parents of AGYW (*n* = 62)	
	n	%	n	%
**Age (years)**				
Median [IQR]	20 [19–20]		42.5 [35–50]	
**Province**				
Central	12	14	20	32
Copperbelt	19	22	16	26
Eastern	18	21	10	16
Luapula	10	11	8	13
Lusaka	28	32	8	13
Education*				
Primary school	3	3	21	35
Secondary/equivalent	77	90	25	42
College or higher	4	5	11	18
Vocational	2	2	3	5
**Civil status**				
Married	6	7	46	74
Unmarried with regular partner	65	75	1	1
Unmarried, no regular partner	16	18	1	1
Separated/divorced/widowed	–	–	14	24
**Sex**				
Female	87	100	47	76
Male	–	–	15	24
**Ever used PrEP?**	44	51	Not asked	Not asked
**Currently using contraception**	53	61	Not asked	Not asked
**Current contraceptive method^**			Not asked	Not asked
Male condoms	24	45		
Injectable	18	34		
Oral contraceptives	8	15		
Implant	5	9		
Other	1	1		
**Own a smartphone or tablet**	43	49	29	47
**Ever accessed health information from internet**				
Yes (of *n* = 29 with personal smart phone)			14	48

*Percentages of only those who responded were calculated

^Multiple method responses permitted

### PrEP product awareness, preferences, and preferred service delivery locations

Many AGYW participants and only some parents of AGYW were aware of oral PrEP, with greater awareness among those in urban provinces. Most AGYW and almost all parents had not heard of the PrEP ring or CAB PrEP. After learning about these methods, most AGYW said they preferred CAB PrEP over the ring or oral PrEP because the injectable could be used discreetly without their parents’ knowledge. In addition, they liked that CAB PrEP has the longest (two months) duration of action, can be used by both men and women, has high efficacy, and protects a person across a range of exposures and not for vaginal sex only (compared to the ring). The small number of participants who preferred the ring also cited discreet use as their main reason, noting that use could be hidden from boyfriends and parents. Very few preferred oral PrEP. The main challenges discussed were that the pill cannot be taken discreetly, and others may think they are living with HIV if they are seen with the pill bottle or taking oral PrEP. Participants said that given different personal preferences, it is important to offer a range of PrEP method choices.

AGYW participants across all provinces cited DREAMS centers, clinics, and hospitals as the locations where they would prefer to receive PrEP services. AGYW participants liked the DREAMS centers because they offer faster service than hospitals or clinics, more convenient locations, a youth-centered service, and staff treat clients with care, explain things well, and have time for clients. Those preferring a clinic or hospital said they liked that products are free of charge and staff are knowledgeable, well-trained, and treat clients well.

### AGYW perspectives: parental engagement in daughters’ use of contraceptives or HIV prevention methods

AGYW participants across all three geographical groups said few parents would be supportive of their children using PrEP. They explained that many parents would not support PrEP use because it would mean their children were engaging in sex. We found no differences in perspectives between AGYWs who had ever used PrEP and those who had never used it.

AGYW participants who thought that their parents would be supportive said that although it is hard to speak with parents about PrEP, disclosing use is the best approach, in part so that parents can provide help if needed:

Me, I think they [parents] would be comfortable knowing what I’m up to instead of finding out from somewhere else because where I come from, transparency is more important than anything else. They will get upset, yes, but the fact that it’s the truth, it will make them comfortable, because whatever happens, they will say we knew about it. There are side effects to these things [referring to PrEP]. Sometimes you take them and you go to the hospital, and sometimes you need someone from home. So, who do you call when something happens to you? (AGYW, Eastern Province)

However, given the perception that many parents would not support PrEP use, most AGYW said it would be difficult for them to speak with their parents about PrEP and contraception specifically and HIV prevention generally. That is why many stated they would hide PrEP use from their parents and would prefer PrEP methods such as CAB PrEP that can be used discreetly:

I wouldn’t be safe because maybe someone at home can see the drugs [oral PrEP]. But the injectable [CAB PrEP] will be a secret for me; even when going to have the service, I can just say I have gone to the market, and then I receive the injection [CAB PrEP] and I wait for two months. What if my parents found me taking the drugs [oral PrEP]? I wouldn’t have any explanation to give them. (AGYW, Central Province)

Many AGYW participants across all provinces said their parents do not play a role in their decisions to use contraceptives or HIV prevention methods and they cannot discuss these topics with their parents. Reasons for their inability to discuss these topics included parents disapproving of daughters engaging in sexual activities before marriage, wanting daughters to finish their education before having children, and believing mistakenly that using contraception before initiating childbearing could result in infertility. However, a few participants said their parents were open to talking to them about contraceptives and HIV prevention method use, mainly citing mothers who supported their daughters and provided advice on using these products.

One AGYW participant said parents’ guidance to their children, especially daughters, is vital to protect them from HIV and pregnancy:

They [parents] have a big role in our lives, especially the girl child. As we grow, they need to guide us on how we should protect ourselves from HIV/AIDS and pregnancy prevention. The first teachers are the parents; they are key to initiate the education, then we will listen to others. (AGYW, Copperbelt Province)

### Parents’ perspectives: initiating and discussing HIV prevention and PrEP use with daughters

Most parents said few of their peers would spontaneously initiate or discuss HIV prevention (except abstinence) and PrEP use with their daughters. Many parents acknowledged difficulty initiating or participating in conversations about PrEP due to strong cultural and religious beliefs in their communities about abstinence for daughters before marriage. Some parents would initiate these discussions only if they discovered their children were at risk of HIV exposure. A few parents said that if they discussed SRH issues with their children, the message would be about abstinence.

A small number of mothers said they advocate for their daughters to use contraception and PrEP and have taken the initiative to discuss these issues with their daughters. One mother described taking her daughter to a clinic for SRH services because she did not want her child to go through the same hardships that she had encountered in her youth:

Me, I got pregnant when I was in grade seven. After I gave birth, everything about school went wrong; hence, I stopped. Then later on, I started school and reached grade nine, but by then, I had already lost interest in school. So I was telling my mother that at school, people used to laugh at me because I had a child. Hence, I would love that my children are taken for family planning to prevent early pregnancies … I wouldn’t want my child to go through the same. (Parent of AGYW, Lusaka Province)

A few fathers said community norms dictate that mothers should speak to their daughters and fathers to their sons about SRH issues, as this father explained:

Because we men feel shy to talk to a girl child, I just can’t sit her down and start telling her about such thing [HIV prevention methods]. That’s why I said I would tell the women [to speak with their daughters]. But if it’s a boy, I would just sit him down and talk to him openly and ask him if they’ve learnt anything about HIV at school and give him the available preventive measures. (Parent of AGYW, Copperbelt Province)

One parent said that the threat of HIV is real, and if children are already sexually active, parents need to be pragmatic and proactively engage in PrEP use conversations with their children:

We just have to accept that our children will use whatever prevention they feel because HIV is real…and these children are indulging in sexual behaviors. So, the parents just have to accept the truth. Even if we say no, they’ll sneak out. And so, whether you like it or not, we just have to educate them on which one is effective. (Parent of AGYW, Luapula Province)

Some parents requested that youth-friendly programs such as DREAMS conduct programs on HIV prevention for youth so they can send their children to those programs rather than to discuss the issue. These parents felt that discussing HIV prevention, PrEP, and other SRH issues with their children would incorrectly imply approval of premarital sex.

Other parents said they would like to speak to their daughters about HIV prevention and PrEP but did not have sufficient knowledge to do so and needed guidance on how to speak with their children about these topics:

So we as parents, we need to learn how to talk to our children. You know, teaching a child does not necessarily mean sending them to do certain things, so us as parents need to be taught on how to talk to our children and how to teach them. (Parent of AGYW, Lusaka Province)

### Parents’ recommendations: support they need to discuss HIV prevention with their children

Parents identified two types of support they need. First, they want knowledge about HIV, what behaviors lead to exposure, and prevention options, including PrEP and the variety of PrEP methods as they become available. Second, parents want to be taught how to discuss these sensitive issues with their children so their children can open up to them and feel supported.

Parents also said it is essential to train more people to sensitize community members, starting with parents, so that they can discuss HIV prevention with their children without worry of censure from others. Some participants mentioned that the DREAMS program was able to conduct these activities:

We should stop being shy with the children; we have to sit down with them. DREAMS helped us to get rid of the awkwardness so that we sit down with the female children and their friends who have good morals and also to teach our fellow neighbors, that will move us forward. (Parent of AGYW, Central Province)

Others suggested sending PrEP information via phones, as the Ministry of Health has done with tuberculosis and malaria information. Participants also said sensitizing communities can be done through radio, television, churches, schools, and youth groups. Community volunteers could conduct door-to-door campaigns and sensitize the community about PrEP through dramas at marketplaces. Community members could also be trained through women’s groups and churches to disseminate HIV prevention information in their communities.

One participant emphasized that messages for parents and for youths should be the same so that the content is not contradictory:

Yeah, I think we as parents will appreciate that the information is reaching us and is also reaching our children. But again, those disseminating also need to take care of the packaging. How the information is packaged, so that it doesn’t conflict with what we also teach our children. So the information needs to be age specific, so that even as we talk to our children, there are no such questions whereby you say, “No, I didn’t see that on TV,” or “I didn’t hear that on radio,” or “The teacher was wrong to have said that.” This should be done so [we] don’t confuse the children.” (Parent of AGYW, Luapula Province)

## Discussion

This research explored awareness of and preferences for PrEP methods in the product pipeline among AGYW and parents of AGYW, as well as AGYW–parent engagement on HIV prevention and SRH. AGYWs in our study overwhelmingly preferred CAB PrEP over oral PrEP or the ring. These results, which highlight the need to prioritize a highly effective, long-lasting method that can be used discreetly without male partners’ and parents’ knowledge, are similar to findings among AGYW in single and multisite studies in Botswana, Kenya, South Africa, and Uganda [21, 22, 33–36]. AGYW’s service delivery preferences reflected the advantages offered by adolescent-friendly sites, particularly DREAMS sites where multiple services are co-located, mirroring findings from other contexts [37–39].

Many AGYW reported inability to disclose PrEP use to their parents due to lack of support based on cultural taboos about premarital sex. Yet, these participants reported desiring parental engagement on PrEP and other SRH issues because they value their parents’ support and guidance. Many parent participants expressed their desire to better understand PrEP methods and support their daughters to prevent HIV but felt uncomfortable doing so, partly due to perceived lack of information and engagement skills. AGYW and parents voiced a need for support, guidance, and an enabling environment for discussions about HIV prevention and other SRH issues. Other studies have reported similar findings about adolescents’ and parents’ willingness to engage in discussions about SRH and the challenges to such engagement [40–42]. These studies recommend developing culturally sensitive interventions targeting parents and adolescents. Based on these data from Zambia, we further recommend that interventions address prevailing cultural issues and social norms that impede parents’ engagement with AGYWs on SRH, such as taboos related to premarital sex. Studies currently in process, such as the IMARA-SA trial [43], are testing interventions responsive to these recommendations and may provide an evidence-based model for adaptation and use.

There is limited evidence on the effectiveness of HIV prevention interventions that engage parents and youth in Zambia and the region. A behavioral intervention for the prevention of HIV and depression tested in the *Our Family Our Future* randomized controlled trial in South Africa was found to be highly acceptable and feasible [44]. This intervention involved sequential community-based sessions with discussions, debates, group activities, and role-play on a variety of topics, including communicating about sexual and mental health; family values; and strategies for healthy interactions and problem-solving.

The DREAMS project, implemented in multiple African countries including Zambia, has associated parent/caregiver programs [27, 45] that were positively perceived by participants in our study. We recommend adapting and testing these interventions in settings beyond DREAMS sites to potentially reach a broader group of parents and youth, particularly in rural settings.

AGYW in a study in South Africa reported that following successful engagement, parents and partners provide the most support for continuing PrEP [12, 15]. However, not all AGYW will be able to discuss PrEP use with their parents. Some participants in our study described situations where disclosing PrEP use can lead to negative parental reactions due to perceived stigma and misconceptions about PrEP. Giovenco et al. [12] suggest that PrEP counseling for AGYW should include discussions on whether or not to disclose use to parents and disclosure strategies should they choose to do so.

### Stakeholder consultation discussion

During the July 2022 stakeholder consultation in Lusaka, Zambia that served as an informal method of validating our preliminary results, the audience agreed that the findings on the following topics were consistent with their understanding:

- AGYW awareness of PrEP and new PrEP methods.
- Preferences regarding new PrEP methods, service delivery sites, and reasons for these preferences among AGYW.
- AGYW and parental perspectives on parents’ engagement with their AGYW on HIV prevention and what parents would think of their adolescent children using PrEP.

Stakeholders offered different views and additional context on parents’ ability to initiate conversations about HIV and SRH with their adolescents and how they might respond to or support requested PrEP use. A few stakeholders acknowledged that some social norms are harmful, such as not discussing these issues with adolescents for fear it will make them promiscuous. However, they emphasized positive norms, such as close family relationships, and recommended that programs promote those norms. They also agreed that much of parents’ reluctance to discuss HIV prevention stems from feeling that they do not have sufficient, correct information or the skills to engage their children on these issues with positive actions such as accompanying them to a clinic. Youth delegates noted there will always be a role for peer educators to either guide engagement with parents or act as a third-party source for youth who believe their parents cannot or would not have these discussions.

Most stakeholders endorsed more programming for parents to “rebrand” discussing sensitive issues with young people as desirable and provide training on how to enhance their conversations and relationships with their children. Stakeholders emphasized ensuring that such training include guidance on providing SRH information to adolescents with a level of detail appropriate for their age and stage of development. These observations enrich our findings and should be incorporated as recommendations for model adaptation in similar contexts.

### Limitations

Some limitations should be considered when interpreting these data. First, many parents and caregivers and some AGYW participants were recruited through DREAMS centers or CSOs with specialized programming for youth HIV prevention; therefore, they may have had greater than typical access to PrEP information and services and may not represent the average end user. However, greater access is also a potential advantage because participants’ responses provide insights into desirable program features that can guide adaptation to improve PrEP services.

We confined AGYW participants to those ages 18 years and older, the age limit for providing informed consent to research participation in Zambia. Therefore, we did not address the perspectives of younger adolescents who may be more likely to rely on their parents for information and guidance.

As a qualitative study, our research explored topics deeply rather than across a breadth of participants. Although we endeavored to sample a diverse group of Zambian provinces to probe different perspectives and experiences, a notable gap is not purposively including people with disabilities and their key influencers.

Also, in some interviews, topics were not probed as deeply across all participants or the wrong guide was used. However, we used IDIs to help articulate areas not fully probed in the FGDs, and questions regarding new PrEP methods and service delivery preferences were similar across FGD and IDI interview guides.

## Conclusion

Although our findings show many parents do not play a role in their daughters’ decisions to use HIV prevention methods or contraception and would feel uncomfortable initiating such discussions with their daughters, parents and adolescents do wish to engage with each other on these issues. To foster open, supportive relationships, programs should consider equipping parents with greater SRH, HIV, and PrEP information and engagement skills and should address social norms that impede parents’ engagement with AGYW on these topics. AGYW also need strategies for engaging their parents, potentially through the guidance of trained counselors or peers. Broader community sensitization on HIV prevention and PrEP is needed, particularly as new products are introduced, to create an enabling environment for dialogue between parents and youth by increasing awareness, countering misconceptions, and reducing stigma.

### Electronic supplementary material

Below is the link to the electronic supplementary material.

Supplementary Material 1: Descriptions of the oral PrEP, PrEP ring, and CAB PrEP methods

## Data Availability

The datasets used and/or analyzed are not publicly available due to concerns about participant privacy but may be made available from the corresponding author on reasonable request.

## Abbreviations

AGYW: Adolescent girls and young women
CAB PrEP: Injectable cabotegravir
CSO: Civil society organization
DREAMS: Determined, Resilient, Empowered, AIDS-free, Mentored and Safe
FGD: Focus group discussion
IDI: In-depth interview
PrEP: Pre-exposure prophylaxis
PrEP ring: Dapivirine vaginal ring
SRH: Sexual and reproductive health
