# Supplementary material for: Creating allies: qualitative exploration of young women’s preferences for PrEP methods and parents’ role in PrEP uptake and user support in urban and rural Zambia
Source: BMC Womens Health. 2024 Jan 25;24:71. doi: 10.1186/s12905-024-02913-7 (PMC10809647; doi:10.1186/s12905-024-02913-7)
Supplement: Supplementary file 1 — Supplementary Material 1: Descriptions of the oral PrEP, PrEP ring, and CAB PrEP methods [file 12905_2024_2913_MOESM1_ESM.pdf]

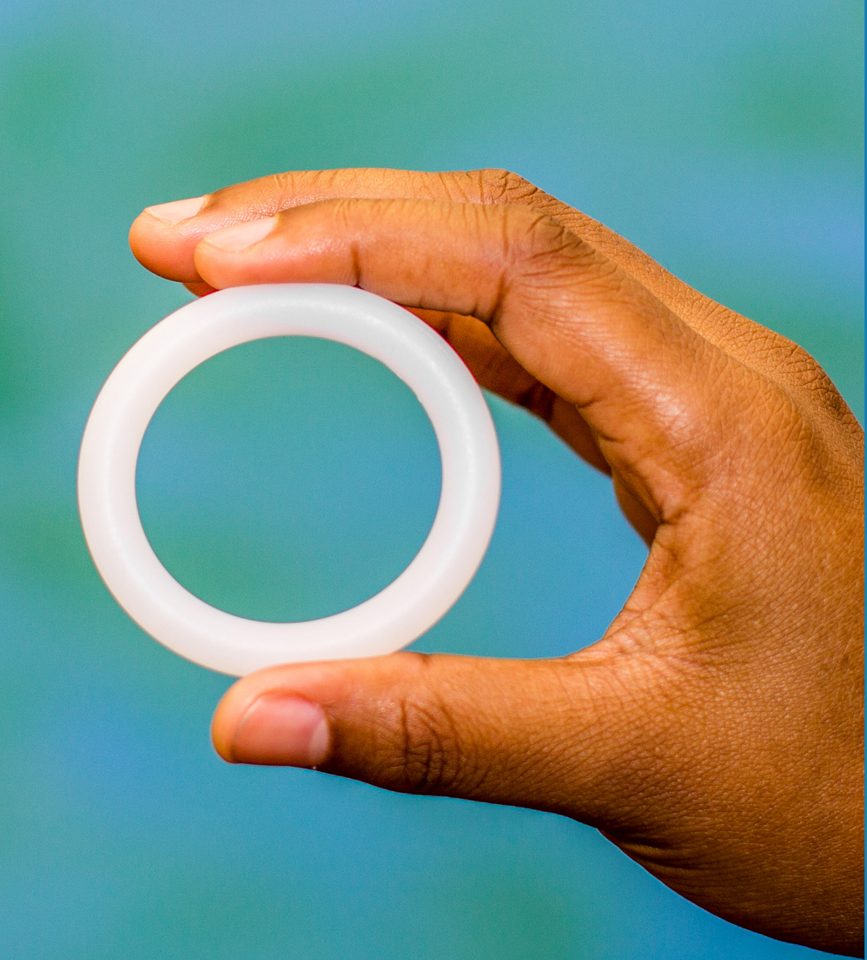

# Dapivirine Vaginal Ring

**“THE RING”**

# Dapivirine Vaginal Ring

## (“the ring”)

- A HIV prevention method for women only.
- A flexible plastic ring that is inserted into the vagina and slowly releases a drug called dapivirine that helps prevent HIV.
- Worn for 28 days, even during menstruation and intimate relations, and then replaced with a new ring.
- Because little of the drug is released into the body, it is less likely to cause side effects or HIV resistance than some other HIV prevention products.
- In March 2021, the World Health Organization included the ring in its updated guidelines as an option for women who cannot or do not wish to use oral PrEP, as part of combination prevention.
- Does not prevent pregnancy or sexually transmitted infections.

**EFFECTIVENESS:**
With consistent use, it has been shown to reduce the risk of HIV infection by 50% or more.

**SIDE EFFECTS:**
May include vaginal discharge or itching, and pelvic or lower abdominal pain. These side effects usually go away within 1–2 weeks without the need to remove the ring.

*The ring has been submitted for review in Zambia by the product developers but has not yet been approved for use.*

USAID FROM THE AMERICAN PEOPLE
PEPFAR
Envision FP

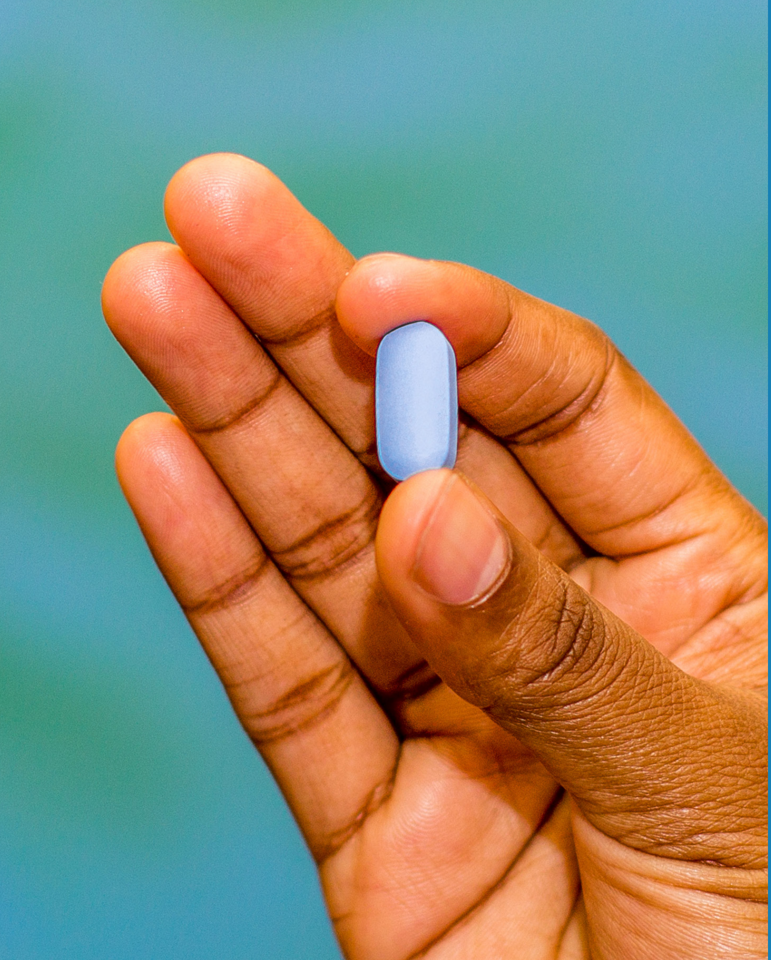

# Oral Pre-Exposure Prophylaxis

**“ORAL PREP”**

# Oral Pre-Exposure Prophylaxis
## (oral PrEP)

- A pill containing drugs such as tenofovir and emtricitabine, or tenofovir and lamivudine, or tenofovir alone, which is taken once daily to prevent HIV.
- For most people, oral PrEP must be taken every day to be effective, but some other ways to use oral PrEP has been studied.
- Does not prevent pregnancy or sexually transmitted infections.

**EFFECTIVENESS:**
Highly effective for all populations, reducing the risk of HIV by about 99% when taken as prescribed.

**SIDE EFFECTS:**
May include nausea, diarrhea, and headaches. These side effects are typically mild and can be managed by a clinician without discontinuing use.

*Approved for use in many countries, including Zambia.*

USAID FROM THE AMERICAN PEOPLE
PEPFAR
Envision FP

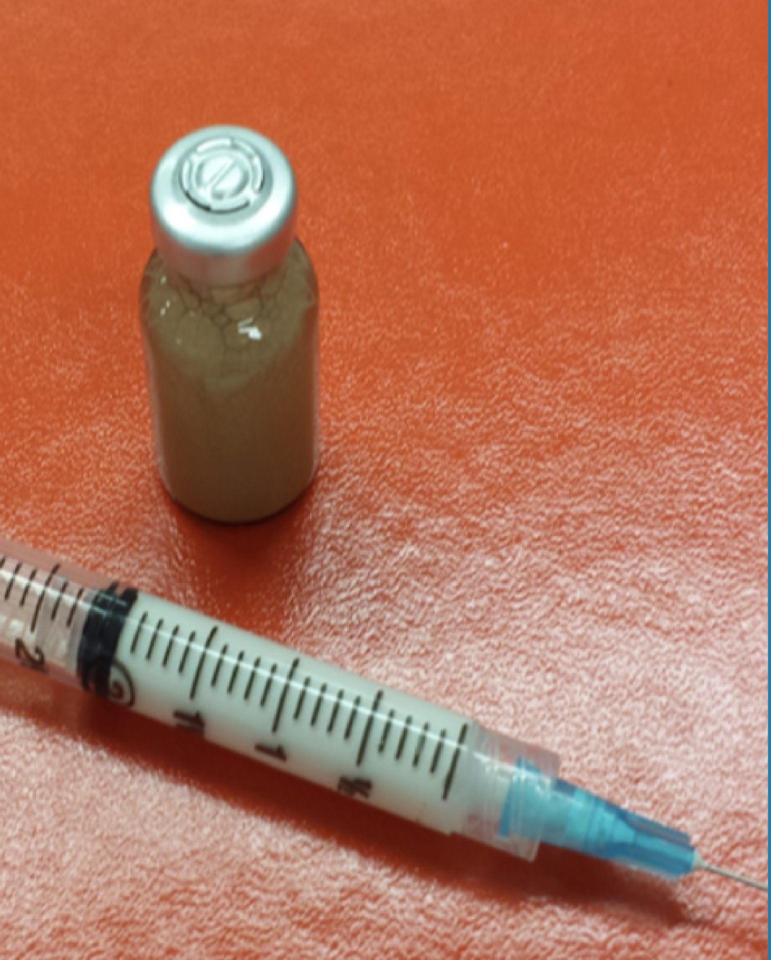

# Long-acting injectable cabotegravir

**“INJECTABLE PREVENTION”**

# Long-acting injectable cabotegravir

## (injectable prevention)

- A long-acting injectable containing cabotegravir, a drug that prevents HIV, called CAB-LA or "injectable prevention".
- Must be injected in a large muscle (like the butt) by a health care provider every two months.
- If a person decides to stop using injectable prevention while still at risk of HIV infection, they need to take daily oral PrEP for up to one year to prevent HIV resistance because low levels of the drug stay in the body for a long time.
- Does not prevent pregnancy or sexually transmitted infections.

**EFFECTIVENESS:**
A recent study found that injectable prevention is more than 99% effective.

**SIDE EFFECTS:**
The most common side effect from injectable prevention noted thus far is soreness or tenderness at the injection site. More will be known about possible side effects as further studies are conducted.

*The injection for HIV prevention has not yet been approved by a global regulatory authority and the earliest it will be available is 2022.*

USAID FROM THE AMERICAN PEOPLE
PEPFAR
Envision FP
